# Transcriptional profiling differences for articular cartilage and repair tissue in equine joint surface lesions

**DOI:** 10.1186/1755-8794-2-60

**Published:** 2009-09-14

**Authors:** Michael J Mienaltowski, Liping Huang, David D Frisbie, C Wayne McIlwraith, Arnold J Stromberg, Arne C Bathke, James N MacLeod

**Affiliations:** 1 University of Kentucky, Department of Veterinary Science, Maxwell H. Gluck Equine Research Center, Lexington, KY, 40546-0099, USA; 2 University of Kentucky, Department of Statistics, 817 Patterson Office Tower, Lexington, KY, 40506-0027, USA; 3 Colorado State University, College of Veterinary Medicine, Gail Holmes Equine Orthopaedic Research Center, Ft. Collins, CO, 80523-1678, USA; 4 University of South Florida, College of Medicine, Department of Orthopaedics & Sports Medicine, 12901 Bruce B. Downs Blvd, MDC Box 11, Tampa, FL, 33612, USA

## Abstract

**Background:**

Full-thickness articular cartilage lesions that reach to the subchondral bone yet are restricted to the chondral compartment usually fill with a fibrocartilage-like repair tissue which is structurally and biomechanically compromised relative to normal articular cartilage. The objective of this study was to evaluate transcriptional differences between chondrocytes of normal articular cartilage and repair tissue cells four months post-microfracture.

**Methods:**

Bilateral one-cm^2 ^full-thickness defects were made in the articular surface of both distal femurs of four adult horses followed by subchondral microfracture. Four months postoperatively, repair tissue from the lesion site and grossly normal articular cartilage from within the same femorotibial joint were collected. Total RNA was isolated from the tissue samples, linearly amplified, and applied to a 9,413-probe set equine-specific cDNA microarray. Eight paired comparisons matched by limb and horse were made with a dye-swap experimental design with validation by histological analyses and quantitative real-time polymerase chain reaction (RT-qPCR).

**Results:**

Statistical analyses revealed 3,327 (35.3%) differentially expressed probe sets. Expression of biomarkers typically associated with normal articular cartilage and fibrocartilage repair tissue corroborate earlier studies. Other changes in gene expression previously unassociated with cartilage repair were also revealed and validated by RT-qPCR.

**Conclusion:**

The magnitude of divergence in transcriptional profiles between normal chondrocytes and the cells that populate repair tissue reveal substantial functional differences between these two cell populations. At the four-month postoperative time point, the relative deficiency within repair tissue of gene transcripts which typically define articular cartilage indicate that while cells occupying the lesion might be of mesenchymal origin, they have not recapitulated differentiation to the chondrogenic phenotype of normal articular chondrocytes.

## Background

Full-thickness articular cartilage defects that penetrate into the subchondral bone undergo a repair process characterized by the in-growth of fibrous tissue within the lesion [1,2]. Initially, blood from the bone marrow below the articular cartilage fills the defect and forms a fibrin clot [2,3]. Subsequent to vascularization of the defect is the proliferation of granulation tissue over the first 10 days as the clot scleroses [2,3]. The granulation tissue is rich in type I collagen fibers and the cells within the tissue have been traced to a mesenchymal origin [2,4-6]. Within full-thickness defects generated by arthrotomy and controlled drilling into the subchondral bone, not more than 30% of total collagen content is type II four months after surgery [4]. Type I fibrillar collagen predominates the extracellular matrix in repair tissue of most full-thickness defects without graft or transplant [4,7]. Decreases in proteoglycan content also occur which render the repair tissue more rigid and unable to fully protect the joint from biomechanical stress [1,4,5,7]. In addition, morphological differences exist between the cells in repair tissue and the chondrocytes of skeletally mature articular cartilage [3]. Repair tissue anchors incompletely to the surrounding articular cartilage matrix adjacent to the lesion [2]. While repair tissue seems to be primarily derived from stromal cells of mesenchymal origin, the functional similarity of these cells to articular chondrocytes is not completely described. Repair tissue is often called fibrocartilage or hyaline-like repair cartilage, though it does not necessarily contain an actual chondrocyte cell population.

The engineering of repair tissue cells is widely investigated in an attempt to improve the chondral surface within injured joints. Techniques like microfracture have been developed in an effort to facilitate healing of the articular surface with cells from the subchondral bone [6,8-13]. There is also a focus on manipulating repair tissue, implanted stem cells, and even autologous chondrocyte transplants in an effort to generate more hyaline-like phenotypes [14,15]. Assessment of the similarity of repair tissue to cartilage is typically done by monitoring established matrix biomarkers, such as type I collagen, type II collagen, and aggrecan core protein. Even with the introduction of growth factors or scaffolds of maintenance proteins associated with the chondrocyte phenotype, the repair tissue is still unable to completely restore the structural and biomechanical integrity of the joint surface, consistent with the limited capacity of articular cartilage to heal.

In this study, we used an equine cDNA microarray containing 9,413 probe sets to compare gene expression profiles of grossly normal articular cartilage and repair tissue occupying medial femoral condyle full-thickness defects in the femorotibial joints of skeletally mature horses four months after a microfracture surgical procedure. The hypothesis tested was that the cells occupying repair tissue four months postoperatively are not identical to articular chondrocytes. Consequently, we would expect the transcriptomes of cells from each tissue to have substantial differences, especially with respect to the expression of cartilage matrix biomarkers.

## Methods

### Animals

Articular cartilage defects were made in the axial weightbearing portion of the medial femoral condyles of four adult Quarterhorses (2-3 years) as previously described by Frisbie *et al. *[6,16] within the guidelines set forth in an Institutional Animal Care and Use Committee-approved protocol at Colorado State University. Briefly, one-cm^2 ^full-thickness articular cartilage lesions were arthroscopically made bilaterally which included the removal of the calcified cartilage layer. This was followed by microfracture penetration of the subchondral bone to create perforations with an approximate spacing of 2-3 mm and depth of 3 mm uniformly within the defect site. The horses were maintained for four months in box stalls (3.65 m × 3.65 m) with controlled hand walking. After euthanasia, repair tissue from the lesions and full-thickness grossly normal articular cartilage from within the same joint were collected from each stifle, rinsed in sterile phosphate-buffered saline, snap-frozen in liquid nitrogen, and stored at -80°C.

### Histology

Samples were also collected and prepared for histological analyses as described in Frisbie *et al. *[17]. Briefly, repair tissue and adjacent cartilage were trimmed with a standard bone saw and Exakt bone saw with a diamond chip blade (Exakt Technologies, Oklahoma City, OK, USA), placed into histological cassettes, and then fixed in 10% neutral buffered formalin for a minimum of 2 days. Samples were then applied to 0.1% EDTA/3%HCl decalcification solution (Thermo Scientific Richard-Allan Decalcifying Solution, cat. no. 8340) which was replenished every three days until specimens were decalcified. Specimens were embedded in paraffin and sectioned at 5 μm. Sections were stained with hematoxylin and eosin or with Safranin-O.

### Total RNA Isolation and Linear Amplification

Normal articular cartilage was reduced to powder with a BioPulverizer (BioSpec Products, Bartlesville, OK, USA) under liquid nitrogen and total RNA was isolated as described by MacLeod *et al. *[18,19]. Briefly, total RNA was isolated in a buffer of 4 M guanidinium isothiocyanate, 0.1 M Tris-HCl, 25 mM EDTA (pH 7.5) with 1% (v/v) 2-mercaptoethanol, followed by differential alcohol and salt precipitations and then final purification using QIAGEN RNeasy columns [18-21]. Repair tissue sample sizes were minimal in size (10-50 mg). Repair tissue was placed in QIAzol reagent (QIAGEN), cut into 1-mm^3 ^slices, and total RNA isolated using the QIAGEN RNeasy Lipid Tissue Mini Kit. RNA quantification and quality assessments were performed with a NanoDrop ND-1000 and a BioAnalyzer 2100 (Agilent, Eukaryotic Total RNA Nano Series II). Total RNA (1 μg) from each tissue sample received one round of linear amplification primed with oligo-dT (Invitrogen - SuperScript RNA Amplification System) [22,23]. Two micrograms of amplified RNA were then used as a template to create fluorescent dye-coupled single-stranded aminoallyl-cDNA probes (Invitrogen - Superscript Indirect cDNA Labeling System, Molecular Probes - Alexa Fluor 555 and 647 Reactive Dyes). For each sample, probes were coupled to both Alexa Fluor dyes individually so that a dye swap comparison could be made.

### Transcriptional Profiling

Microarray slides were printed with clones selected from a cDNA library generated using equine articular cartilage mRNA from a 15-month old Thoroughbred [24]. Microarray slides were pre- hybridized in 20% formamide, 5× Denhardt's, 6× SSC, 0.1% SDS, and 25 μg/ml tRNA for 45 minutes. The slides were then washed five times in deionized water, once in isopropanol, and spun dry at 700 g for 3 minutes [25]. Two labeled cDNA samples, one repair tissue and the other normal cartilage from the same joint, were combined with 1× hybridization buffer (Ambion, 1× Slide Hybridization Buffer #1, cat. no. 8801), incubated for 2 minutes at 95°C, and then applied to the slide under a glass lifterslip for 48 hours at 42°C. All hybridizations were performed in duplicate with a dye swap to eliminate possible dye bias [26]. Sequential post-hybridization washes were each for 5 minutes as follows: 1× SSC, 0.2% SDS at 42°C; 0.1× SSC, 0.2% SDS at room temperature; and twice with 0.1× SSC at room temperature. The slides were then spun dry under argon gas at 700 g for 3 minutes. Each slide was coated once in DyeSaver 2 (Genisphere) and allowed to dry for 10 minutes. Slides were scanned using a GenePix 4100A scanner and spot intensities were computed using GENEPIX 6.0 image analysis software (Axon Instruments/Molecular Devices).

### Statistics and Analysis

Raw mean intensity data for each probe set pair of all the microarray scans were statistically analyzed by planned linear contrast [27] using SAS (SAS Institute, Cary, NC). One sample t-tests were performed, which were followed by a Benjamini-Hochberg correction based on a false discovery rate of 2.2% for probe sets with a p-value < 0.01 [28]. Differences in the transcriptional profiling data between repair tissue and articular cartilage were analyzed based on a linear model formulation with fixed tissue and dye effect, and random chip, horse, and leg effect. A dye swap design was used, so for each available leg, the sum of the two measurements corresponding to the articular cartilage was subtracted from the sum of the measurements corresponding to the repair tissue. Each intensity measurement (*I*) is modeled statistically as:



with the components designated as follows: *d*, additive effects due to dye (red or green); *c*, chip effect (1-16); *t*, tissue (repair or normal); *h*, horse (1-4); *l*, leg (left or right); *E*, statistical error. The dye swap design yields two outcomes per location and tissue type. Thus, for each of the eight locations corresponding to a particular leg of a particular horse, a new aggregated quantity is calculated that takes into account all measurements related to this location. The only remaining systematic effect represents the expressional difference between tissues with remaining statistical error. Since there were 4 horses with 2 femorotibial joints per horse, eight such tissue differences were evaluated. Gene identity was assigned for each microarray ID from an internal annotation database through selection of either the best RNA RefSeq BLAST (*E *< 1 × 10^-7^) or Protein RefSeq BLAST (*E *< 1 × 10^-5^) result [29-31]. Gene ontology (GO) annotation was derived from batch queries of the Database for Annotation, Visualization, and Integrated Discovery (DAVID) Bioinformatics tool or manually through individual NCBI Entrez Gene queries [32,33]. The human ortholog of each gene was predicted and used for the determination of overrepresentation of GO categories via Expression Analysis Systematic Explorer (EASE) standalone software [32,34]. Statistical data, fold change quantities, and GO annotations were managed within an Excel spreadsheet (Microsoft, Redmond, WA). Microarray data are available at the NCBI Gene Expression Omnibus (GEO) under Series Accession GSE11760.

### Validation of Microarray Hybridization Results with RT-qPCR

Differential expression for selected genes was validated using quantitative polymerase chain reactions (RT-qPCR). Briefly, total RNA was reverse-transcribed into cDNA using an oligo-dT primer with the Promega Reverse Transcription System (Promega, cat. no. A3500). Quantitative "real-time" PCR (7500 Sequence Detection and 7900 HT Fast Real-Time PCR Systems, Applied Biosystems, Foster City, CA) was performed using TaqMan Gene Expression Master Mix (Applied Biosystems) and intron-spanning primer/probe sets (Assays-by-Design, Applied Biosystems) designed from equine genomic sequence data (Ensembl - ; UCSC Genome Browser - ). Beta-2-microglobulin (B2M) and large ribosomal protein P0 (RPLP0) were selected as endogenous control transcripts because they showed the greatest stability for the sample set as defined by the geNorm reference gene application (data not shown) [35]. Steady state levels of mRNA encoding type I procollagen (COL1A2), type II procollagen (COL2A1), cartilage oligomeric matrix protein (COMP), dermatopontin (DPT), fibroblast activation protein (FAP), and tenascin-C (TNC) were selected for validation (Table 1). Amplification efficiencies were measured by the default fit option of LinRegPCR while maintaining the cycle threshold as a data point within the measured regression line [36]. Since amplification efficiencies for some of the genes were determined to be different between sample groups (normal and repair) by paired t-test, mean group efficiencies were utilized for adjustment of results for each gene (data not shown) [37]. Relative expression levels of target genes were normalized to the relative quantities of endogenous control genes using geometric averaging with the geNorm VBA applet [35]. For each gene of interest, mean fold change was determined by first finding the difference in transcript abundance between normal and repair samples from each leg and then by determining the mean difference amongst all legs for that gene. Statistical analysis of RT-qPCR results was performed with a general linear model (GLM) strategy using SPSS software with consideration for variables of horse, leg, and tissue. One-tail (α = 0.05) test of the hypothesis that microarray data are valid was considered for tissue effect, which is the significance reported. By the same GLM analyses, no significant "within horse" leg effect was demonstrated for any of the genes validated (data not shown).

**Table 1:** Primer nucleotide sequences used in RT-qPCR assays for genes described in the study.

**Gene Name**	**Gene Symbol**	**Forward Primer**	**Reverse Primer**
Beta-2-microglobulin	B2M	5-CGGGCTACTCTCCCTGACT-3	5-GTGACGTGAGTAAACCTGAACCTT-3

Ribosomal protein, large, P0	RPLP0	5-CTGATTACACCTTCCCACTTGCT-3	5-AGCCACAAATGCAGATGGATCA-3

Procollagen, type I, alpha 2	COL1A2	5-TGAGACTTAGCCACCCAGAGT-3	5-GCATCCATAGTGCATCCTTGATTAGG-3

Procollagen, type II, alpha 1	COL2A1	5-CTGGCTTCAAAGGCGAACAAG-3	5-GCACCTCTTTTGCCTTCTTCAC-3

Cartilage oligomeric matrix protein	COMP	5-CGAGCCCGGCATCCA-3	5-CCCAGGGCCTGTGGAG-3

Dermatopontin	DPT	5-GGAGATCAACAGGGCTGGAAT-3	5-CCGCCACCAGTCCATTGTT-3

Fibroblast activation protein	FAP	5-AGACTATCTTCTCATCCACGGAACA-3	5-CCGGATATGCCGTGGTTCTG-3

Tenascin-C	TNC	5-TCAGCCATCACTACCAAGTTCAC-3	5-GAACCTCAGTAGCAGTCAAATCTCT-3

## Results

### Repair tissue histology

**Figure 1:**
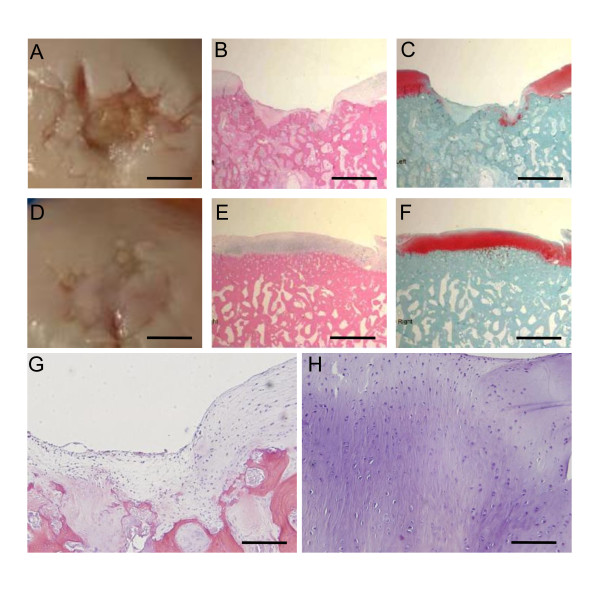
**Gross and histological assessment of repair tissue**. At the four month post-operative time point, gross assessment of the repair tissue indicated variations in the healing response (A, D). Moreover, the repair tissue was not fully congruent with the surrounding articular cartilage within each lesion (B, E). Histologically, variations in the repair resulted in elongated and flattened cells as observed with H&E staining (G, 10×) surrounded by variable levels of proteoglycan-deficient matrix as assessed by Safranin-O staining (C, F). The interface between repair tissue and surrounding cartilage is evident (H, 20×). Representative lesions depicted are from Horse 4 (Left: A, B, C, G; Right: D, E, F, H). Scale bar: 5 mm in A, D; 2.50 mm in B, C, E, F; 250 microns in G; 125 microns in H.

Tissue samples were harvested four months after surgical induction of full-thickness cartilage lesion with microfracture. Gross examination revealed repair tissue within each lesion that was dimpled in appearance and not completely level with the articular surface (Figure 1A, D). Histologically, repair tissue generally had homogeneous matrix architecture with elongated, flattened cells (Figure 1G) that interfaced with surrounding articular cartilage (Figure 1H). Varying levels of repair tissue were noted with some lesions having a poor response (Figure 1A-C), while others appeared to respond better (Figure 1D-F). Safranin-O staining demonstrated that the repair tissue was generally proteoglycan-deficient relative to the adjacent normal articular cartilage surrounding the lesions (Figure 1C), but there was variation with some repair tissue samples showing evidence of proteoglycan content (Figure 1F).

### Overall level of differential gene expression

**Figure 2:**
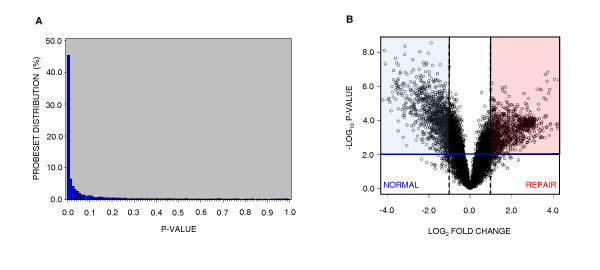
**Microarray profiling data distributions**. The distribution of p-values for differential gene expression comparing articular cartilage to repair tissue using 8 replicates (4 left legs and 4 right legs from 4 horses) is demonstrated in a histogram (A); the column at the far left are those differentially expressed at p < 0.01 and represents 4269 probe sets, or 45.6% of the microarray. (B) A volcano plot illustrates the divergent distribution of probe sets between normal articular cartilage and repair tissue. Log_2_-fold change differences of transcript abundance are represented across the horizontal axis; fold changes greater than 2-fold are located outside of the vertical dotted lines. Significance (-log_10 _p-value) is represented on the y-axis with p < 0.01 located above the blue horizontal line. Probe sets within the blue shaded region have a fold change ≥ 2 at p < 0.01 in normal articular cartilage relative to repair tissue. Probe sets within red shaded region have a fold change ≥ 2 at p < 0.01 in repair tissue relative to normal articular cartilage.

**Figure 3:**
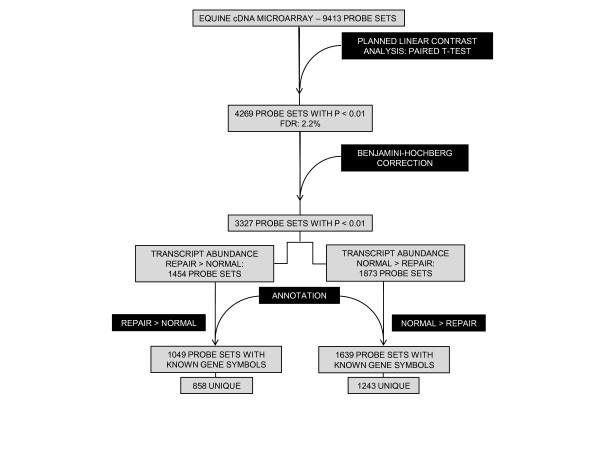
**Flowchart of cDNA microarray data analysis**. Expression data were initially analyzed by planned linear contrast with a Benjamini-Hochberg correction yielding 35.3% of the probe sets on the microarray demonstrating significant differential gene expression (p < 0.01). Of these, 43.7% and 56.3% of the probe sets represented increased relative transcript abundance for repair tissue > normal cartilage and normal cartilage > repair tissue, respectively. When annotation is applied to these probe sets, 2688 (80.8%) have known gene symbols with a redundancy across this subset of probe sets equal to 21.8% yielding 2101 unique gene symbols.

A total of 4,269 probe sets (45.4%) were differentially expressed (p < 0.01; Figure 2A). A clear transcriptome divergence was evident between the two tissue types (Figure 2B). After Benjamini-Hochberg correction, 3,327 (35.3%) significant probe sets remained (Figure 3). Of these probe sets, 1,454 demonstrated greater transcript abundance in repair tissue relative to grossly normal articular cartilage, and 1,873 demonstrated greater transcript abundance in normal articular cartilage relative to repair tissue. Assessment of probe set annotation produced 2,688 significant probe sets with known gene identities. Correcting for redundancy where different probe sets hybridize to the same mRNA transcript yielded 2,101 unique gene symbols. Of these, 858 gene symbols were present at higher steady-state levels in repair tissue and are designated *repair *> *normal*, while 1,243 of the gene symbols are designated *normal *> *repair *(Figure 3).

### Ontological differences

When significant probes are organized according to molecular function ontology with a fold change threshold of two, ontological categories of differentially abundant transcripts emerge by EASE analyses (Tables 2 and 3). Categories statistically overrepresented in normal articular cartilage include skeletal development and glycosaminoglycan binding, which contain many of the conventional cartilage biomarkers (Table 2). Protease and endopeptidase inhibitor activities were also overrepresented for normal cartilage; these categories contain matrix metalloproteinase inhibitors essential to the maintenance of cartilage matrix (Table 2). In contrast, the categories statistically overrepresented in repair tissue (immune response, cytoskeletal and cell component organization, and histogenesis) are indicative of wound healing or tissue re-modeling (Table 3). One shared overrepresented category is calcium ion binding, which is involved with protein-folding conformation of matrix molecules and chondrocyte differentiation, among other functions. A second overrepresented category common to the two tissue types was extracellular matrix component. This result is quite plausible since extracellular matrix (ECM) plays an essential role in defining the phenotypes of both tissues. Functional differences are demonstrated by individual transcript abundance for ECM components involved in processes such as fibrillogenesis and proteoglycan synthesis which are further delineated below.

**Table 2:** Overrepresented ontological categories for transcripts with >2-fold difference in normal articular cartilage versus repair tissue.

**GO System**	**Gene Category**	**List Hits**	**List Total**	**Population Hits**	**Population Total**	**EASE Score**
Cellular Component	extracellular	69	380	244	2556	2.01E-08

Biological Process	skeletal development	21	379	55	2575	0.00005

Molecular Function	receptor activity	53	385	218	2611	0.00012

Cellular Component	extracellular matrix	30	380	100	2556	0.00015

Biological Process	development	82	379	394	2575	0.00036

Molecular Function	signal transducer activity	81	385	393	2611	0.00059

Molecular Function	calcium ion binding	34	385	134	2611	0.00129

Biological Process	regulation of transcription (3)	82	379	410	2575	0.00134

Molecular Function	glycosaminoglycan binding	12	385	31	2611	0.00318

Molecular Function	endopeptidase inhibitor activity (2)	11	385	27	2611	0.00341

Molecular Function	protease inhibitor activity	11	385	27	2611	0.00341

Cellular Component	membrane (2)	148	380	842	2556	0.00553

Molecular Function	steroid hormone receptor activity	7	385	13	2611	0.00668

Biological Process	transcription	83	379	441	2575	0.00764

Molecular Function	insulin-like growth factor binding	6	385	10	2611	0.00893

Redundant or similar categories were removed. When present, the number of redundant or similar categories are indicated in parentheses within the listed "gene category." "GO System" represents the major gene ontological system (*i.e.*, cellular component, biological process, or molecular function). "Gene category" shows a descriptive term shared by a group of genes. "List hits" are the numbers of differentially abundant transcripts that belong to the gene category. "List total" is the number of differentially expressed genes within the corresponding cellular component, biological process, or molecular function system. "Population hits" represent the total number of genes found on the microarray possessing that specific gene category annotation (*e.g.*, extracellular, skeletal development, etc.). "Population Total" represents the total number of genes found on the microarray possessing that ontological system annotation. "EASE score" is a measure of overrepresentation that scales the results of a statistical analysis (Fisher's exact test) by biasing against categories supported by few genes.

**Table 3:** Overrepresented ontological categories for transcripts with >2-fold difference in repair tissue versus normal articular cartilage.

**GO System**	**Gene Category**	**List Hits**	**List Total**	**Population Hits**	**Population Total**	**EASE Score**
Biological Process	immune response	23	270	98	2575	0.00032

Biological Process	defense response	23	270	103	2575	0.00066

Cellular Component	cytoskeleton	39	275	216	2556	0.00095

Cellular Component	actin cytoskeleton	18	275	73	2556	0.00133

Biological Process	response to biotic stimulus	26	270	130	2575	0.00148

Biological Process	organelle organization and biogenesis	21	270	96	2575	0.00162

Biological Process	cytoskeleton organization and biogenesis	16	270	65	2575	0.00216

Cellular Component	extracellular matrix	21	275	100	2556	0.00368

Biological Process	histogenesis	7	270	16	2575	0.00398

Biological Process	cell motility	18	270	84	2575	0.00492

Biological Process	response to external stimulus	36	270	220	2575	0.00569

Molecular Function	calcium ion binding	25	279	134	2611	0.00627

Biological Process	protein complex assembly	10	270	34	2575	0.00646

Biological Process	cell adhesion (3)	26	270	145	2575	0.00685

Molecular Function	hydrogen ion transporter activity (2)	13	279	53	2611	0.00808

Redundant or similar categories were removed. When present, the number of redundant or similar categories are indicated in parentheses within the listed "gene category." "GO System" represents the major gene ontological system (*i.e.*, cellular component, biological process, or molecular function). "Gene category" shows a descriptive term shared by a group of genes. "List hits" are the numbers of differentially abundant transcripts that belong to the gene category. "List total" is the number of differentially expressed genes within the corresponding cellular component, biological process, or molecular function system. "Population hits" represent the total number of genes found on the microarray possessing that specific gene category annotation (*e.g.*, extracellular, skeletal development, etc.). "Population Total" represents the total number of genes found on the microarray possessing that ontological system annotation. "EASE score" is a measure of overrepresentation that scales the results of a statistical analysis (Fisher's exact test) by biasing against categories supported by few genes.

### Individual genes

**Figure 4:**
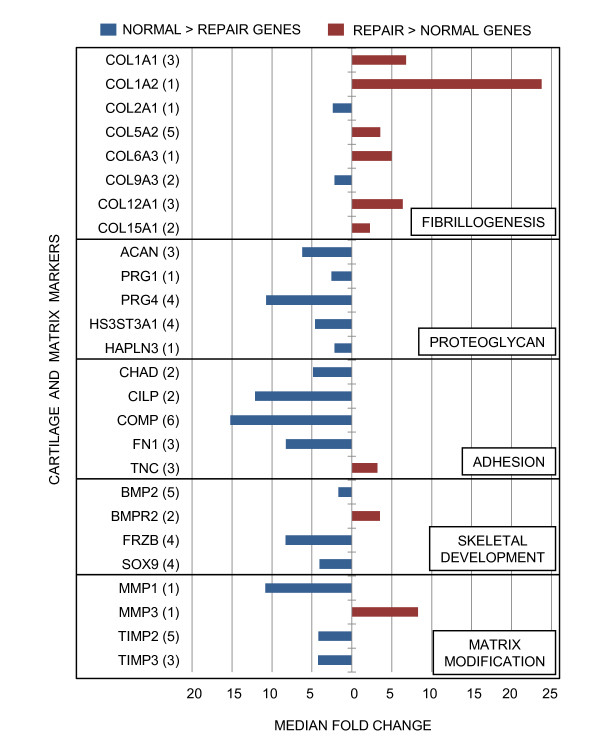
**Microarray transcriptional profiles of articular cartilage and repair tissue molecules/biomarkers**. Bars represent median fold changes of differentially expressed genes (p < 0.01) previously associated with cartilage and fibrocartilage. Gene symbols are organized by functional annotation and are listed with the number of representative probe sets in parentheses.

Expression differences for genes encoding biomarkers typically associated with normal articular cartilage and repair tissue corroborate previous reported findings (Figure 4). That is, transcript abundance for collagen types II and IX were greater in normal articular cartilage relative to repair tissue. The expression of type I collagen and several type-I-associated collagen types (V, VI, XII, XV) were up-regulated in repair tissue relative to normal articular cartilage. Moreover, transcript abundance was greater in normal cartilage for proteoglycans, an associated sulfotransferase, non-collagenous adhesion proteins, and skeletal development biomarkers linked to cartilage development. In contrast, transcripts were up-regulated in repair tissue for Tenascin- C and matrix metalloproteinase 3, which are both associated with wound healing.

**Figure 5:**
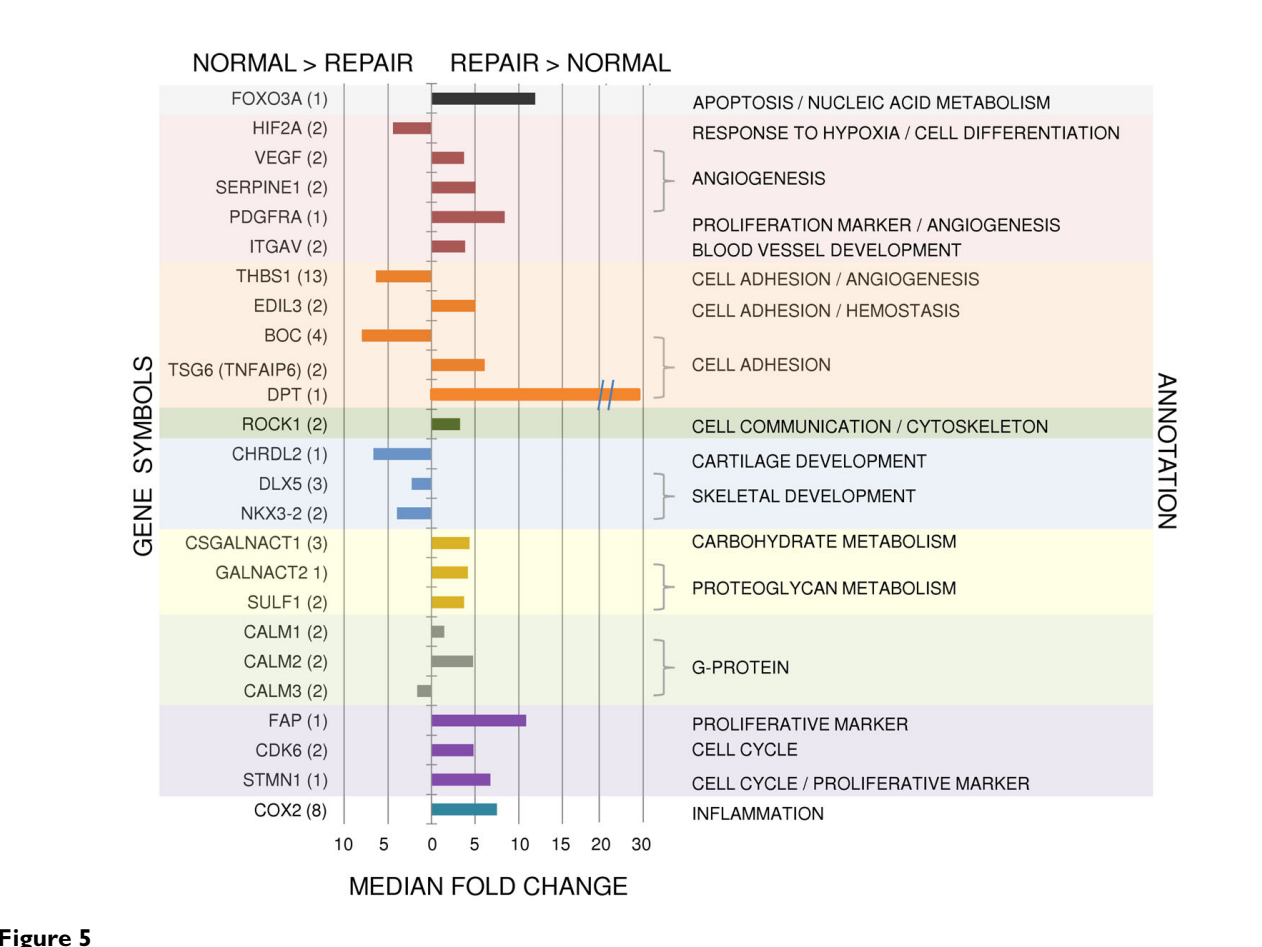
**Microarray transcriptional profiles for representative genes of interest**. Median fold changes are shown for differentially expressed genes (p < 0.01) which further distinguish normal articular cartilage and repair tissue. Individual genes are organized by molecular function. Gene symbols are given with the number of representative probe sets in parentheses.

Other genes with limited or no established functional annotation in chondrocytes were also differentially expressed between normal articular cartilage and repair tissue. Within the angiogenesis category, transcripts encoding vascular endothelial growth factor and the serpin peptidase inhibitor SERPINE1 had higher steady state levels in repair tissue (Figure 5). Also represented were genes involved in cell adhesion, cell communication, skeletal development, and carbohydrate and proteoglycan metabolism. Of note was increased transcript abundance in repair tissue for proliferative cell markers like fibroblast activation protein (FAP) and stathmin-1, as well as the inflammatory mediator cyclooxygenase-2 (COX2) (Figure 5).

### Quantitative PCR validation

**Figure 6:**
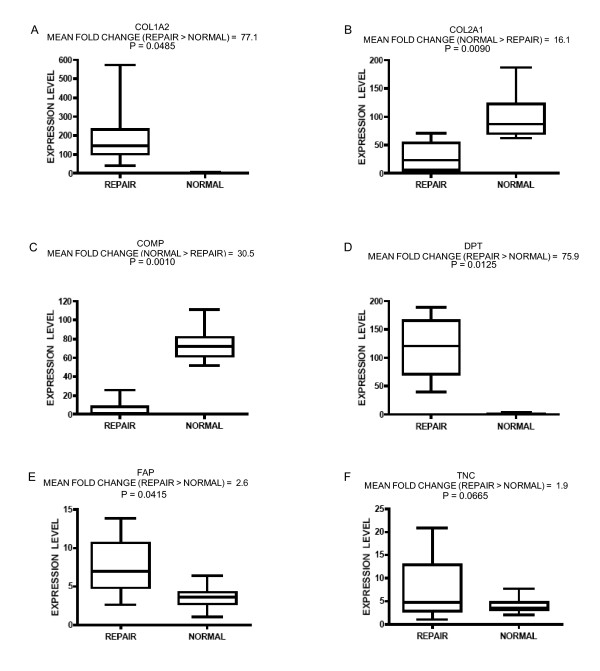
**RT-qPCR validation of differential gene expression**. Significant up-regulation of COL2A1 (B) and COMP (C) gene expression in normal articular cartilage relative to repair tissue was confirmed. Higher steady-state levels of COL1A2 (A), DPT (D), and FAP (E) transcripts in repair tissue were also confirmed. Gene expression of TNC (F) demonstrates a trend of increased steady state abundance in repair tissue relative to normal articular cartilage, though statistical significance was not achieved. Steady state mRNA levels for each gene were standardized to the sample with the lowest value. Plots are depicted as box and whisker plots demonstrating the median (solid line), upper and lower quartiles, and highest and lowest values (range bars). Mean fold differences are given above the box and whisker plots for each gene; one-tailed general linear model (α = 0.05) statistical analysis applied with SPSS software.

Steady-state transcript abundance was measured for endogenous controls beta-2-microglobulin (B2M) and large ribosomal protein P0 (RPLP0), as well as target genes type I procollagen alpha-2 chain (COL1A2), type II procollagen alpha 1 chain (COL2A1), cartilage oligomeric matrix protein (COMP), dermatopontin (DPT), fibroblast activation protein (FAP), and tenascin-C (TNC). Relative quantification of target transcripts revealed significant increases in mRNA abundance for COL2A1 and COMP in normal articular cartilage (Figure 6). Fold change differences were similar or slightly greater than what was measured by microarray profiles. Increased COL1A2, DPT, and FAP transcript abundance for repair tissue was also validated by RT-qPCR (Figure 6). Transcript abundance for TNC in repair tissue demonstrated an increasing trend by RT-qPCR, though significance was not achieved (Figure 6).

## Discussion

Histological analyses and transcriptional studies identified clear differences between chondrocytes of grossly normal articular cartilage and the cells present in repair tissue of full-thickness articular lesions following a microfracture surgical procedure. At four months post-surgery, repair tissue is morphologically discernible from normal cartilage. Type I collagen transcripts are detected in the repair tissue, and much of the repair tissue is proteoglycan-deficient. Moreover, a substantial transcriptional divergence is readily apparent between the two cell types even at a genomic level. Analyses of overrepresented gene categories for differentially expressed transcripts demonstrate broad functional differences.

Conventional biomarker transcripts used to characterize a chondrocytic phenotype indicated that the repair tissues in this sample set were quite different from the adjacent articular cartilage in the same joint. Increased transcript levels for types II and IX collagen were found in the articular cartilage (Figure 4). Quantitative RT-PCR indicated a 16.1-fold expression difference for COL2A1 in articular cartilage relative to repair tissue (p = 0.0090, Figure 6B). In contrast, abundance of transcripts associated with type I collagen-rich fibrous tissues were greater in repair tissue (Figure 4). Steady-state mRNA levels for COL1A2 were 77.1-fold higher in repair tissue relative to articular cartilage (p = 0.0485, Figure 6A). These transcriptional data directly support published biochemical results which demonstrated differing collagen type I: type II ratios for articular repair tissue and perilesional articular cartilage through detection of cleaved peptides [4,7]. Differences in the magnitude of fold changes in microarray and RT-qPCR results can be explained by the differences in dynamic range of detection between hybridization-based assays and amplification-based assays [38]. Notable differences for proteoglycans between repair tissue and the surrounding articular cartilage were observed with transcript levels and by Safranin-O staining (Figures 1, 4). Proteoglycan differences have also been noted through Safranin-O staining of articular repair tissue in the distal femur of the New Zealand White rabbit [2] and in the distal radial carpal bone of the horse [7], relative to proteoglycan content of perilesional articular cartilage in both studies.

Divergent characteristics between articular cartilage and repair tissue extend to transcripts of other matrix proteins. Transcripts encoding cartilage macromolecules believed to play a role in cell-cell and cell-matrix interactions were significantly less abundant in repair tissue relative to normal articular cartilage (Figure 4). Such transcripts included chondroadherin (CHAD), cartilage intermediate layer protein (CILP), cartilage oligomeric matrix protein (COMP), and fibronectin (FN1) [39-44]. COMP interacts with type II collagen for fibrillogenesis and has been shown to bind to the chondroitin sulfate glycosaminoglycans associated with aggrecan. COMP expression is initially up-regulated in chondrocytes exposed to increased dynamic compression [45,46], those from the superficial zone in fibrillated OA cartilage [47], and chondrocytes adjacent to an OA lesion [44]; however, transcript levels in repair tissue at the four month time point were 30.5-fold lower (p = 0.0010, Figure 6C). Matrix molecules like CILP which are present in normal cartilage slow down the responsiveness of chondrocytes to insulin-like growth factor 1 (IGF-1) as a result of accumulation of calcium pyrophosphate dehydrate [43]. Thus, CILP might inhibit the ability of the surrounding chondrocytes to expand and occupy the lesion [43]. Transcript abundance for hypoxia inducible transcription factor 2α (HIF-2α) was up-regulated in normal cartilage (Figure 5) and has been found to support the cartilage phenotype by (SRY-box 9) SOX9 induction of matrix genes [48]. In contrast, tenascin-C (TNC), which is typically found in provisional matrices throughout development and wound healing [49-52], demonstrated greater transcript levels in repair tissue by microarray analyses (Figure 4). While statistical significance was not confirmed by RT-qPCR (p = 0.0665, Figure 6F), upregulation of TNC has been noted in early stages of osteoarthritis and also during the repair process of many other tissues through *in situ *hybridization, immunohistochemistry, and knockout mouse studies [49,53-55]. Based on its function in the expansion of provisional matrices, it is likely that analyses of earlier time points would have detected greater divergence of TNC mRNA levels.

Within the repair tissue, differential expression was noted for transcripts encoding proteins involved in wound healing and matrix synthesis. Shapiro *et al. *have shown that stromal cells of mesenchymal origin from the subchondral bone enter into the wound with the blood which fills the full-thickness lesion [2]. With angiogenic cues such as vascular endothelial growth factor (Figure 5) and vascularization from the subchondral bone, these cells proliferate within the granulation tissue to occupy the lesion [2,56,57]. Increased transcript abundance of fibroblast activation protein (FAP) is consistent with the proliferative cellular response reported by Shapiro *et al. *(Figure 5) [2]. RT-qPCR indicated a 2.6-fold relative expression difference for FAP in repair tissue four months post-microfracture relative to articular cartilage (p = 0.0415, Figure 6E). Assessment of FAP expression at additional time points during the repair process would further delineate its importance. Steady-state levels of dermatopontin (DPT) were also elevated in repair tissue (Figures 5, 6D). Fibrillogenesis of type I collagen is accelerated by DPT, which has previously been localized in skin fibroblasts, skeletal muscle, heart, lung, bone, and chondrocytes that de-differentiate while expanding in monolayer culture [58-60]. DPT interacts synergistically with decorin and transforming growth factor-β1 to bolster collagen synthesis and accelerate fibrillogenesis to the point of decreasing fibril diameters in proliferating skin fibroblast cultures [59]. A wound healing process is further indicated by the 7.5-fold up-regulation of cyclooxygenase 2 (COX2, Figure 5), an inflammatory modulator shown to be essential in the repair of bone fractures and growth plate lesions [61]. Transcript profiles for COX2 and S100 protein are compatible with chondrogenic differentiation of stromal cells [61,62], but the consistent deficiency of cartilage matrix protein biomarkers highlighted by the switch of type I collagen (COL1A1, COL1A2) in place of type II collagen (COL2A1) as the primary fibrillar collagen document the failure of true hyaline cartilage restoration.

A limitation of this study must be noted. Tissues utilized in these experiments included repair tissue and grossly normal articular cartilage from within the same joint. Thus, any gene expression differences between grossly normal cartilage within the lesioned joint and cartilage from an intact articular surface from another joint were not assessed. Differences have been reported with intact cartilage from human OA joints [63]. However, equine joints used for the current sample set had minimal OA and the defects were freshly created in the medial femoral condyles four months prior to tissue sample collection.

## Conclusion

Transcriptional profiling data support the hypothesis and indicate that repair tissue cells following a microfracture surgical procedure are still very different from normal articular chondrocytes at the four month postoperative time point. The cell and matrix organizational phenotypes of repair tissue are substantially different from those of chondrocytes within mature articular cartilage that has developed and adapted to biomechanical strains from birth. Microarray data in the current study corroborate what has been reported previously at mRNA and protein levels for conventional cartilage biomarkers, but extends our understanding by documenting differences in transcript abundance across multiple ontology categories and genes not previously studied in these tissues. By directing further research toward factors which contribute to the transcriptome dissimilarities of repair tissue and normal articular cartilage phenotypes, we should advance our understanding of the repair process and improve upon therapeutic strategies directed at restoring the structural and biomechanical integrity of the joint surface.

## List of Abbreviations

B2M: beta-2-microglobulin; CHAD: chondroadherin; CILP: cartilage intermediate layer protein; COL1A2: collagen, type 1, alpha 2; COL2A1: collagen, type 2, alpha 1; COMP: cartilage oligomeric matrix protein; COX2: cyclooxygenase-2; DPT: dermatopontin; EASE: Expression Analysis Systematic Explorer; ECM: extracellular matrix; FAP: fibroblast activation protein; GEO: Gene Expression Omnibus; GO: gene ontology; IGF-1: insulin-like growth factor-1; NCBI: National Center for Biotechnology Information; RT-qPCR: quantitative polymerase chain reaction; RPLP0: ribosomal protein, large, P0; SOX9: sex-determining region homeobox-9; TNC: tenascin-C.

## Competing interests

The authors declare that they have no competing interests.

## Authors' contributions

Experimental surgical procedures were performed by CWM and DDF. Tissue sample harvesting was performed by DDF and MJM. Histological techniques were performed by CWM, DDF, and MJM. Molecular biology studies and bioinformatics analyses were performed by MJM and JNM. Statistical analysis of microarray data was performed by LH, ACB, and AJS. Statistical analysis of RT-qPCR data was performed by MJM. All authors contributed to the writing of this manuscript.

## Pre-publication history

The pre-publication history for this paper can be accessed here:


